# No difference in biomarkers of ischemic heart injury and heart failure in patients with COVID-19 who received treatment with chloroquine phosphate and those who did not

**DOI:** 10.1371/journal.pone.0256035

**Published:** 2021-08-16

**Authors:** Josefine Beck-Friis, Susannah Leach, Elmir Omerovic, Rickard Zeijlon, Magnus Gisslen, Aylin Yilmaz

**Affiliations:** 1 Department of Infectious Diseases, Sahlgrenska University Hospital, Gothenburg, Sweden; 2 Department of Microbiology and Immunology, University of Gothenburg, Gothenburg, Sweden; 3 Department of Clinical Pharmacology, Sahlgrenska University Hospital, Gothenburg, Sweden; 4 Department of Cardiology, Sahlgrenska University Hospital, Gothenburg, Sweden; 5 Department of Molecular and Clinical Medicine, Institute of Medicine, University of Gothenburg, Gothenburg, Sweden; 6 Department of Internal Medicine, Sahlgrenska University Hospital, Gothenburg, Sweden; 7 Department of Infectious Diseases, Institute of Biomedicine, University of Gothenburg, Gothenburg, Sweden; Universite de Liege (B34), BELGIUM

## Abstract

**Background:**

Chloroquine was promoted as a COVID-19 therapeutic early in the pandemic. Most countries have since discontinued the use of chloroquine due to lack of evidence of any benefit and the risk of severe adverse events. The primary aim of this study was to examine if administering chloroquine during COVID-19 imposed an increased risk of ischemic heart injury or heart failure.

**Methods:**

Medical records, laboratory findings, and electrocardiograms of patients with COVID-19 who were treated with 500 mg chloroquine phosphate daily and controls not treated with chloroquine were reviewed retrospectively. Controls were matched in age and severity of disease.

**Results:**

We included 20 patients receiving chloroquine (500 mg twice daily) for an average of five days, and 40 controls. The groups were comparable regarding demographics and biochemical analyses including C-reactive protein, thrombocytes, and creatinine. There were no statistically significant differences in cardiac biomarkers or in electrocardiograms. Median troponin T was 10,8 ng/L in the study group and 17.9 ng/L in the control group, whereas median NT-proBNP was 399 ng/L in patients receiving chloroquine and 349 ng/L in the controls.

**Conclusions:**

We found no increased risk of ischemic heart injury or heart failure as a result of administering chloroquine. However, the use of chloroquine to treat COVID-19 outside of clinical trials is not recommended, considering the lack of evidence of its effectiveness, as well as the elevated risk of fatal arrythmias.

## 1. Introduction

Coronavirus disease 2019 (COVID-19) is an infection caused by severe acute respiratory syndrome coronavirus 2 (SARS-CoV-2), with a symptomatology that varies from no (or very mild) symptoms in the upper airways to severe viral pneumonia with respiratory failure. According to current understanding, approximately 80% of symptomatic cases are mild, 15% severe, and 5% critical [1], while the proportion of asymptomatic cases is estimated to be 30% to 40% [2]. The most common complications in patients with severe disease are interstitial pneumonia with respiratory failure, pulmonary thrombosis, arrhythmia, acute cardiac, and acute kidney injury [3, 4]. The mortality among patients in intensive care varies between different studies [4]; in Sweden the mortality among patients requiring invasive ventilation is approximately 20% [5].

As of January 2021, the only drugs approved by the European Medicines Agency (EMA) and US Food and Drug Administration (FDA) are the antiviral drug remdesivir, dexamethasone (EMA only), convalescent plasma (FDA only), the janus kinase inhibitor baricitinib in combination with remdesivir (FDA only), and monoclonal antibodies (FDA only) [6, 7]. Multiple trials ongoing worldwide include new treatment options and drugs that have already been developed for other diseases [8, 9]. One of the drugs that has been widely administered is the antimalarial drug chloroquine, either as chloroquine phosphate or as the less toxic derivative hydroxychloroquine [9]. Because of the immunomodulatory effects of chloroquine, these drugs have long been used to treat inflammatory diseases such as rheumatoid arthritis and systemic lupus erythematosus. Chloroquine has been shown to affect cytokine production [10, 11], which raised hopes that it might mitigate the cytokine release syndrome believed to be associated with severe COVID-19 [12]. Studies have also shown a broad antiviral effect in vitro against several coronaviruses including SARS-CoV-2 [13]. The first positive reports of the clinical efficacy of chloroquine came from China in February 2020 [14], and shortly thereafter a small French study showed a positive effect of chloroquine on symptoms and viral clearance [15]. Since then, chloroquine has been used to a varying degree in many countries, including Sweden. The Department of Infectious Diseases at Sahlgrenska University Hospital, Gothenburg, discontinued the use of chloroquine phosphate at the end of March 2020 due to a lack of evidence regarding efficacy and the risk of severe adverse events. Several studies have since reported severe adverse events arising from the use of chloroquine, with or without the co-administration of azithromycin, to treat COVID-19, especially arrhythmias and QT-prolongation [16, 17]. While the potential benefits of chloroquine/hydroxychloroquine continue to be widely debated, after promising initial reports several clinical trials have been published with no positive results regarding viral clearance and clinical status as compared to standard of care [18]. The hydroxychloroquine arm of the Solidarity Trial, launched by WHO and partners, was permanently halted in June 2020 when data showed that, in comparison to standard of care, hydroxychloroquine did not result in a reduction of in-hospital mortality [19, 20].

To our knowledge, no studies of markers of cardiac injury during chloroquine treatment for COVID-19 have been published to date. The primary aim of our study was to examine whether patients with COVID-19 undergoing treatment with chloroquine phosphate had an increased risk of cardiotoxicity with regard to ischemic heart injury, heart failure, and arrhythmias, when compared to patients receiving standard treatment.

## 2. Methods

### 2.1 Study design and population

This is a retrospective study of patients with COVID-19 who received treatment with chloroquine phosphate (500 mg twice daily) and matched controls from the same time period who did not receive chloroquine phosphate. Inclusion criteria were a) confirmed diagnosis of COVID-19 by positive polymerase chain-reaction test (PCR) from the upper respiratory tract for SARS-CoV-2; b) admission to the Department for Infectious Diseases at Sahlgrenska University Hospital, Gothenburg, Sweden; and c) having received a minimum of two doses of chloroquine phosphate. Exclusion criteria were lack of testing for cardiac biomarkers (cardiac troponin T [cTnT] and N-terminal pro b-type natriuretic peptide [NT-proBNP]), or lack of stored blood samples from either the treatment period or the first four weeks of symptomatic infection. Controls were chosen from the same patient cohort, with chloroquine phosphate treatment as the exclusion criteria. Participants were matched as closely as possible for severity of disease and age.

### 2.2 Data collection and variables of the study

We collected data regarding sex, age, weight, height, comorbidities, medication, date of symptom onset, severity of disease, electrocardiogram (ECG) results, laboratory values, and mortality. Severity of disease was divided into mild (no oxygen therapy required), moderate (oxygen therapy administered through nasal canula or face mask), and severe (need of high-flow oxygen and/or ventilator). Laboratory values of interest were creatinine, estimated glomerular filtration rate (eGFR), potassium, C-reactive protein (CRP), blood lymphocytes, total leukocyte count, neutrophils, platelet count, cTnT, and NT-proBNP. Variables related to chloroquine treatment included date and length of treatment, and drug dosage (daily and total values).

For patients whose cardiac markers had not been analyzed during treatment or within 12 hours after their last dose of chloroquine, biobanked serum was sent for analysis of cTnT and NT-proBNP. Laboratory values from the control group and study group were matched as closely as possible with regard to time between onset of symptoms and collection of blood samples. Serum samples in the biobank that matched more closely in time were analyzed for cardiac markers. Definitions of heart failure (not likely, possible, or probable) were based on age-stratified reference levels from the Department of Clinical Chemistry at Sahlgrenska University Hospital with NT-proBNP < 300 (age 17 to 49 years), or < 400 (age > 49 years) indicating heart failure unlikely; NT-proBNP 300 to 400 (age 17 to 49 years), or 400 to 900 (age 50 to 75 years), or 400 to 1800 (age ≥ 76 years) indicating heart failure possible; and NT-proBNP > 400 (age 17 to 49 years), or > 900 (age 50 to 75 years), or > 1800 (age ≥ 76 years) indicating heart failure probable.

For patients who had an ECG performed during treatment or within two weeks of their last dose of chloroquine, a specialist in cardiology analyzed the ECGs focusing on arrhythmias and signs of heart dysfunction. When ECGs before and during or after treatment were available, they were analyzed and compared. For controls, ECGs taken as closely in time as possible to the cardiac markers were preferred; ECGs taken more than four weeks after onset of symptoms were excluded.

### 2.3 Statistics

Data was analyzed with IBM SPSS Statistics version 26, Stata software (version 16.1, StataCorp, College Station, Texas, USA), and R (version 4.1.0, R Core Team, 2020). Figures were produced using the R package ggplot2. Continuous variables are presented as median (I–III quartile), and categorical variables as absolute numbers (percentages). For comparisons between the two groups, the Mann Whitney U-test was used for continuous variables and the Fischer’s exact test for categorical variables. *P*-values less than 0.05 were considered statistically significant. We evaluated the relative importance of all variables from Tables 1–3, and the additional variables start of chloroquine treatment, admission to intensive care unit, day of blood test, activated partial thromboplastin time (APTT), international normalized ratio (INR), procalcitonin, magnesium, d-dimer, heart rate, heart rhythm, and normal QTc-time, for occurrence of significant cTnT leakage. Relative variable importance was calculated using a generalized boosted regression model as implemented in the R package gbm. Generalized boosted regression modelling is a highly efficient machine-learning method that handles a large number of observations and predictors and produces a prediction model in the form of an ensemble of weak prediction models. Typically, the decision trees allow for complex modeling of interactions and non-linear functions between predictors.

**Table 1:** Demographic characteristics of 60 study participants.

	*Total* (n = 60)	*Chloroquine* (n = 20)	*Controls* (n = 40)
Age; median (IQR)	60 (50–72)	61 (51–72)	60 (50–70)
Gender; no (%)			
Male	49 (82)	17 (85)	32 (80)
Female	11 (18)	3 (15)	8 (20)
Body Mass Index; median (IQR)	29 (26–32)^a^	29 (27–31)^b^	29 (26–32)^c^
Comorbidities, number; median (IQR)	1 (0–3)	0 (0–3)	1 (1–3)
Previous heart disease; no (%)	12 (20)	5^d^ (25)	7^e^ (18)
Ischemic heart disease; no (%)	6 (10)	1 (5)	5 (13)
Cardiovascular risk factors; no (%)^f^	34 (57)	9 (45)	25 (63)
Other drugs affecting QTc time; no (%)	5 (8)	2 (10)	3 (8)
Severity of disease; no (%)			
Mild	3 (5)	1 (5)	2 (5)
Moderate	12 (20)	4 (20)	8 (20)
Severe	45 (75)	15 (75)	30 (75)
Ventilator; no (%)	36 (60)	13 (65)	23 (58)
Deceased; no (%)	11 (18)	3 (15)	8 (20)
Chloroquine treatment length, days; median (IQR)	5 (3–8)	5 (3–8)	0
Total dosage chloroquine, mg (IQR)	5250 (3000–7500)	5250 (3000–7500)	0

IQR = interquartile range.

^a^ Data available for 42 patients.

^b^ Data available for 14 patients.

^c^ Data available 28 patients.

^d^ Cardiac arrhythmia 4, valvular heart disease 1, ischemic heart disease 1.

^e^ Cardiac arrhythmia 3, valvular heart disease 1, ischemic heart disease 5, congestive heart failure 1.

^f^ Hypertension, diabetes, dyslipidemia.

**Table 2:** Laboratory findings for study participants.

	*Total* (n = 60)	*Chloroquine* (n = 20)	*Controls* (n = 40)	*P-value*
Onset of symptoms to blood test (days); median (IQR)	13 (10–15)	14 (10–15)	12 (10–14)	0.231
Troponin T, ng/L; median (IQR)	14.4 (8.0–47.3)	10.8 (7.4–22.8)	17.9 (8.0–55.1)	0.111
NT-proBNP, ng/L; median (IQR)	349 (132–1000)	399 (143–1235)	349 (130–765)	0.784
eGFR, mL/min/1.73m^2^	77^a^ (59–71)	76 (67–86)	78^b^ (58–92)	0.848
Creatinine, μmol/L; median (IQR)	78^a^ (71–102)	86 (74–94)	77^b^ (71–105)	0.749
Potassium, mmol/L; median (IQR)	4.0^a^ (3.7–4.3)	4.2 (3.9–4.4)	4.0^b^ (3.7–4.2)	0.365
CRP, mg/L; median (IQR)	135^c^ (73–260)	180 (63–300)	125^d^ (79–188)	0.399
White blood cell count, 10^9^/L; median (IQR)	7.8 (5.8–10.1)	7.5 (5.8–9.5)	8.3 (6.0–10.3)	0.351
B-lymphocytes, 10^9^/L; median (IQR)	1.0 (0.8–1.3)	1.1 (0.9–1.3)	1.0 (0.8–1.2)	0.162
Neutrophils, 10^9^/L; median (IQR)	6.4 (4.5–8.7)	5.5 (4.2–7.9)	6.8 (4.7–8.8)	0.154
Thrombocytes, 10^9^/L; median (IQR)	240^e^ (166–363)	271 (216–385)	230^f^ (162–347)	0.152

IQR = interquartile range, eGFR = estimated glomerular filtration rate, CRP = C-reactive protein, NT-proBNP = N-terminal prohormone of brain natriuretic peptide.

^a^ Data available for 59 patients.

^b^ Data available for 39 patients.

^c^ Data available for 58 patients.

^d^ Data available for 38 patients.

^e^ Data available for 54 patients.

^f^ Data available for 34 patients.

**Table 3:** ECG analyses of 41 participants.

	*Total* (n = 41)	*Chloroquine* (n = 10)	*Controls* (n = 31)	*P-value*
QTc-time ≥ 460 ms; no (%)	9 (22)	4 (40)	5 (16)	0.185
QTc-time ≥ 480 ms; no (%)	4 (10)	1 (10)	3 (10)	1.000
ST-segment abnormality	3 (7)	0	3 (10) ^a^	0.564
T-wave inversion	18 (44)	2 (20)	16 (52)	0.142

ECG = electrocardiogram, QTc = corrected QT interval, ms = milliseconds.

^a^ ST-segment depression 2, ST-segment elevation 1.

The study was approved by the Swedish Ethics Review Authority (Registration number 2020–01771). Patients were included after written informed consent to use data from their medical records and samples in research was obtained at the time of admission to the hospital. All data were retrospectively collected between April and August 2020. The data was not anonymized when compiled by the authors, but was anonymized before statistical analysis.

## 3. Results

In the six weeks between 26 February and 15 April 2020, a total of 226 patients were admitted to the Department of Infectious Diseases, Sahlgrenska University Hospital with a diagnosis of COVID-19. Twenty-five of them were administered chloroquine, five of whom were excluded from our study because their cardiac markers had not been analyzed or no stored serum samples were available from their treatment period. The remaining 20 patients comprised the chloroquine arm. Forty matched controls who had not undergone chloroquine treatment were also included.

The groups were demographically similar, with a median (interquartile range [IQR]) age of 61 (51–72) years in the study group, compared with a median age (IQR) of 60 (50–70) years in the control group (Table 1). In the study group there were 17 (85%) men, while there were 32 (80%) men in the control group. The median body mass index (BMI) was 29 in both groups. Five (25%) patients in the study group and eight (18%) in the control group had a history of heart disease. Ischemic heart disease was present in one (14%) patient with elevated cTnT in the study group, whereas four patients (57%) had a cardiovascular risk factor (diabetes, hypertension and/or hyperlipidemia). In the control group there were four (17%) patients with elevated cTnT and ischemic heart disease, and 16 (70%) patients with a cardiovascular risk factor. Two (10%) patients in the study group and three (8%) in the control group were taking medication known to potentially affect QT-interval (2 alfuzosin, 2 escitalopram, 1 tacrolimus). There were similar rates of disease severity in both groups: 5% with mild, 20% with moderate, and 75% with severe COVID-19. Among patients receiving chloroquine, 13 (65%) required invasive mechanical ventilation compared to 23 (58%) of the controls. The overall in-hospital mortality rate was 18% (*n* = 11) with three deaths occurring in the study group and eight in the control group.

Median (IQR) duration of treatment with chloroquine was 5 (3–8) days, and the median total dosage was 5250 (3000–7500) mg. Time between onset of symptoms and first day of treatment was a median (IQR) of 10 (8–13) days.

**Fig 1:**
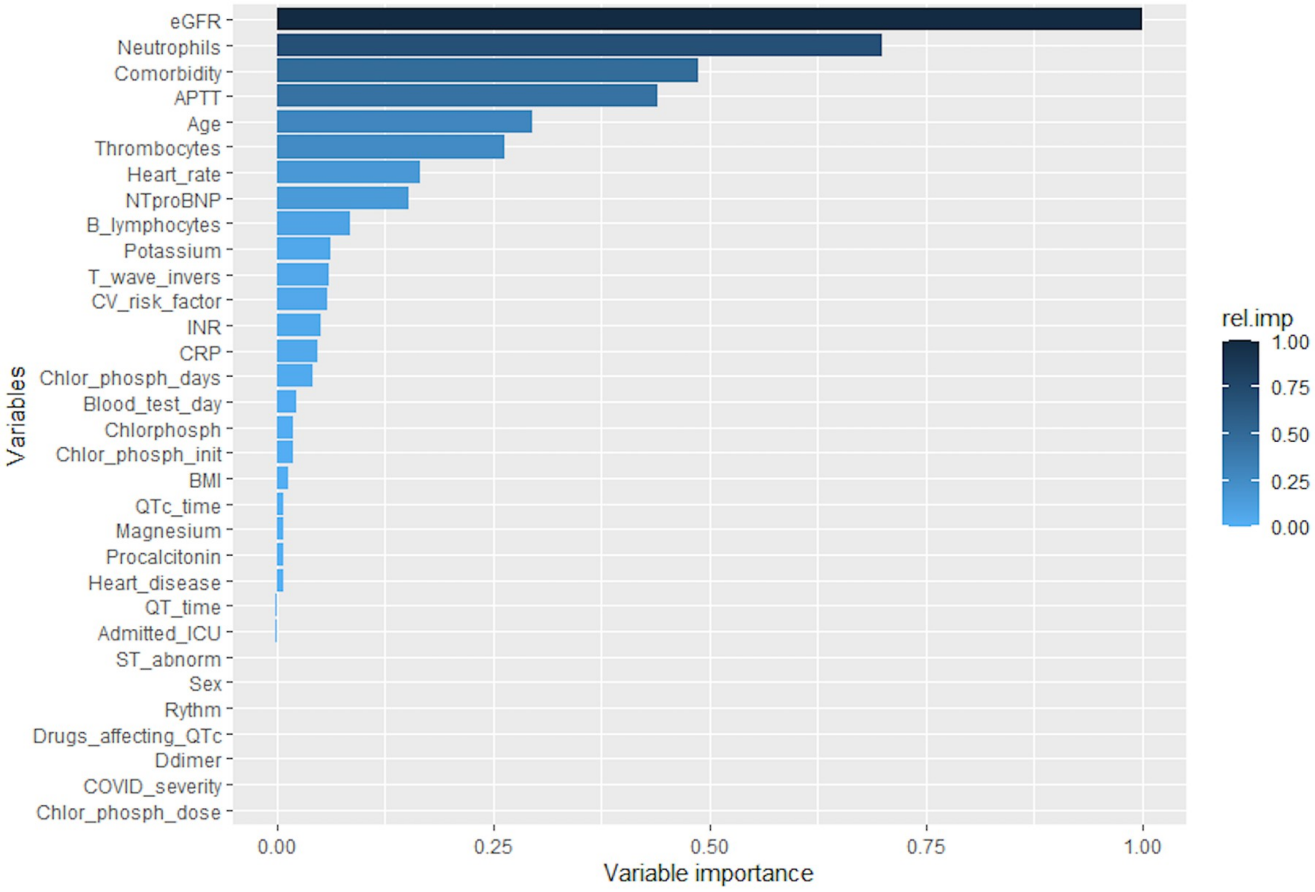
Relative importance of predictors of cardiovascular involvement. eGFR = estimated glomerular filtration rate, comorbidity = numbers of comorbidities, APTT = activated partial thromboplastin time, INR = international normalized ratio, T_wave_invers = T-wave inversion, Chlor_phosph_days = number of days with chloroquine phosphate, CV_risk_factor = cardiovascular risk factors (hypertension, diabetes, dyslipidemia), Chlor_phosph_init = days since symptom onset when chloroquine phosphate was initiated, Blood_test_day = day since symptom onset when blood samples were collected, BMI = body mass index, Chlorphosph = treatment with chloroquine phosphate, Heart_disease = type of previous heart disease specified (cardiac arrhythmia 7, valvular heart disease 2, ischemic heart disease 6, congestive heart failure 1), Chlor_phosph_dose = total dose of chloroquine phosphate. Missing data: eGFR 1, APTT 21, thrombocytes 6, heart rate 19, INR 21, potassium 1, T wave inversion 19, CRP 2, BMI 18, magnesium 34, procalcitonin 34, QTc time 19, QT time 19, d-dimer 33, ST abnormalities 19, rhythm 19. Relative variable importance was calculated using a generalized boosted regression model.

The details of the biochemical analyses are presented in Table 2. Median (IQR) time between symptom onset and sampling of cardiac biomarkers was 14 (10–15) days in the study group and 12 (10–14) days in the control group. All other biochemical variables were recorded as closely as possible in time to that of the cardiac markers. There were no statistically significant differences in any of the variables analyzed. For patients receiving chloroquine, cTnT levels were in median (IQR) 10.8 (7.4–22.8) ng/L, and 17.9 (8.0–55.1) ng/L for the controls. Seven (35%) patients in the study group and 23 (58%) controls had elevated cTnT (> 13.9 ng/L). Fig 1 shows the relative importance of several variables in predicting elevation in cTnT. Treatment with chloroquine had a low relative importance, whereas eGFR, neutrophils, comorbidity, and APPT had high relative importance on cTnT levels.

Median (IQR) NT-proBNP was 399 (143–1235) ng/L in the study group and 349 (130–765) ng/L in the control group. Ten (50%) patients had NT-proBNP values that did not indicate heart failure, and 10 (50%) patents in the study group had possible or probable heart failure. There were 23 (58%) patents with no heart failure, and 17 (43%) patents with possible or probable heart failure, in the control group.

ECGs were performed in ten patients on chloroquine treatment and in 31 controls (Table 3). There were no significant differences in QTc interval prolongation, ST-segment abnormalities, or T-wave inversion. Three patients had their ECGs performed before and after initiation of chloroquine. In one of these patients, QTc interval went from normal to pathologic, increasing from 456 milliseconds (ms) to 473 ms. In the other two, the QTc interval remained the same in one and increased by five ms in the other patient, none of the increases being pathologic. Abnormalities in ST-segment were not present in any of the ECGs. However, T-wave inversion appeared in two patients after initiation of treatment.

## 4. Discussion

We have examined biomarkers of ischemic heart injury and heart failure in patients with varying severities of COVID-19 who either received or did not receive treatment with chloroquine phosphate. In this small study population, we found no significant differences in cTnT or NT-proBNP levels between patients who received chloroquine phosphate and those who did not, indicating that chloroquine had no general effect on cardiac biomarkers.

The COVID-19 outbreak is a global pandemic with considerable mortality. Effective and well-tolerated treatments are therefore urgently needed. The old anti-malarial drug chloroquine phosphate was one of the first pharmaceuticals to be widely used against COVID-19, based on positive preliminary reports from China and France [12, 15]. It was immediately adopted in most countries, including Sweden. However, doubts were soon raised about the safety of the drug. The Department of Infectious Diseases at Sahlgrenska University Hospital was among the first clinics to stop using chloroquine at the end of March 2020, because of the lack of evidence that it was effective and its potential for causing severe adverse events. In early April, a small French study [21] could not demonstrate the positive effects on viral clearance that had been reported earlier [15]. Doubts about using chloroquine to treat COVID-19 patients were soon raised because of reports of severe adverse events and inconclusive data on clinical effects [22]. Descriptions of the adverse effects of chloroquine became more frequent and several countries discontinued its use as a standard treatment for COVID-19. There were at least 11 active (recruiting or not-yet recruiting) clinical trials of chloroquine as a treatment for COVID-19 (prophylactic or treatment of active disease) registered at clinicaltrials.gov on 18 June 2021 [23]. However, several clinical trials were halted as the positive preliminary reports of its efficacy failed to be confirmed in several studies [18, 20]. Chloroquine treatment of COVID-19 is no longer supported by most clinicians. Nevertheless, it may well be of interest in future pandemics/virus outbreaks given that it has had historical interest for other infectious diseases such as SARS [24], influenza [25], yellow fever [26], and HIV [27].

To the best of our knowledge, ours is the first study to analyze markers of heart failure and ischemic injury in patients treated with chloroquine phosphate for COVID-19. A pre-pandemic review from 2018 reported that 8/127 patients receiving long-term treatment with hydroxychloroquine and/or chloroquine had elevated cTnT, and 21/46 patients had an ejection fraction less than 40%, indicating mild-to-severe heart failure [28]. In our study, we could find no increased risk of elevated cTnT among patients who received chloroquine phosphate as compared to those who did not, and the slightly elevated NT-proBNP levels indicating possible or probable heart failure (50% in the study group versus 43% in the control group) were not statistically significant. Moreover, our analyses of ECGs showed no significant differences in signs of ischemic heart injury. Treatment duration in the pre-pandemic study was approximately seven to eight years longer than in our study, and the risk of heart failure may increase with long-term treatment. Thus far, there is no evidence of increased risk for ischemic heart injury during short-term treatment with chloroquine phosphate, and the risk for ischemic injury over long-term treatment appears to be only slightly elevated.

Several studies have shown an increased risk of cardiovascular events during lower respiratory tract infections [29, 30], and specifically during infection with SARS-CoV-2 [3, 31], a finding supported by the high number of patients with elevated cTnT and NT-proBNP in both groups in our study. In a machine learning-based analysis of predictors of elevated cTnT, we found that chloroquine had a low relative importance, whereas factors such as kidney function, neutrophils, comorbidity, and APTT had high relative importance. Age and thrombocytes were of moderate relative importance. Elevated creatinine at admission was found to be a predictor of myocardial injury in COVID-19 in another retrospective analysis [32], and studies have shown that patients with chronic kidney disease generally have higher cTnT levels [33]. Increased APTT, coagulopathy, inflammation, and advanced age have previously been shown to be associated with myocardial injury during COVID-19 infection [34, 35]. Risk factors for cardiovascular disease were present in most patients with elevated cTnT levels, a finding supported by a previous study in which myocardial injury was seen to be significantly more common in COVID-19 patients with chronic coronary syndromes than in patients without [36]. Therefore, while it is difficult to distinguish the cause of myocardial injury among our participants, it is recommendable that chloroquine be used with caution specifically in patients with previous cardiovascular disease, impaired kidney function, and advanced age, until further research has been done.

The pharmacological effects of chloroquine phosphate in cardiac toxicity that have been recognized thus far involve an impact on the electrophysical properties of the heart, such as blocking potassium channels, which in turn could cause drug-induced long QT-syndrome [9]. In the pre-pandemic review cited earlier [28], electrophysical conduction disorders were reported in 85% of patients receiving hydroxychloroquine and/or chloroquine for various inflammatory and infectious diseases. Of 91 patients with COVID-19 receiving hydroxychloroquine and azithromycin, 23% had significant QTc prolongation in a retrospective study [37]. This has been confirmed in further studies, that included individuals with suspected torsade de pointes [16]. Researchers studying patients with COVID-19 receiving hydroxychloroquine as monotherapy found that 19% developed prolonged QTc of 500 ms and 3% had an increase in QTc—time of 60 ms [17]. A retrospective study of 397 patients with COVID-19 treated with chloroquine [38] showed that the QTc interval gradually increased during the treatment period, probably due to the drug’s long half-life of 1.6 days. On the contrary, a multicenter study of 649 COVID-19 patients receiving hydroxychloroquine found only a modest QTc prolongation [39]. Factors identified as predictors of prolonged QTc-time during treatment with the combination hydroxychloroquine and azithromycin include increased troponin, older age, congestive heart failure, and higher admission creatinine [40]. In our study, where ECGs unfortunately were only available for 41 patients, we could not see any significant differences in QTc prolongation or signs of ischemic heart injury when comparing patients with and without chloroquine treatment. However, one of the three patients with a baseline ECG, and a second ECG taken while treated for COVID-19 developed a pathologic QTc prolongation, indicating a potential risk of severe arrythmia in using chloroquine to treat COVID-19.

Our study has several limitations. One major limitation is the small sample size, which makes statistical comparisons uncertain. As data was collected retrospectively, we were unable to measure serum concentrations of chloroquine phosphate. Since there was no systematic ECG collection, we did not have baseline and follow-up ECGs for most of the patients studied.

## 5. Conclusions

We found no differences in acute ischemic heart injury or heart failure in our relatively small sample of hospitalized patients with COVID-19, whether they received treatment with chloroquine phosphate or not. ECG findings from the even smaller number of patients who had ECGs performed showed no differences regarding QTc prolongation or signs of ischemic heart injury. However, in the absence of large-scale studies, chloroquine treatment for COVID-19 infection cannot be considered safe with regard to myocardial infarction and heart failure. Therefore, it cannot be recommended at the present time that chloroquine be used to treat COVID-19 outside of clinical trials, considering the lack of evidence of its efficacy and the elevated risk it presents for arrythmia and sudden cardiac death.

## References

[1] Wu Z, McGoogan JM. Characteristics of and Important Lessons From the Coronavirus Disease 2019 (COVID-19) Outbreak in China: Summary of a Report of 72314 Cases From the Chinese Center for Disease Control and Prevention. JAMA. 2020;323(13):1239–42. doi: 10.1001/jama.2020.2648 32091533

[2] Oran DP, Topol EJ. Prevalence of Asymptomatic SARS-CoV-2 Infection: A Narrative Review. Ann Intern Med. 2020;173(5):362–7. doi: 10.7326/M20-3012 32491919 PMC7281624

[3] Wang D, Hu B, Hu C, Zhu F, Liu X, Zhang J, et al. Clinical Characteristics of 138 Hospitalized Patients With 2019 Novel Coronavirus-Infected Pneumonia in Wuhan, China. JAMA. 2020;323(11):1061–9. doi: 10.1001/jama.2020.1585 32031570 PMC7042881

[4] Phua J, Weng L, Ling L, Egi M, Lim CM, Divatia JV, et al. Intensive care management of coronavirus disease 2019 (COVID-19): challenges and recommendations. Lancet Respir Med. 2020;8(5):506–17. doi: 10.1016/S2213-2600(20)30161-2 32272080 PMC7198848

[5] Intensivvårdsregistret S. Svenska Intensivvårdsregistret [cited 2020 December 1]. https://www.icuregswe.org/data—resultat/covid-19-i-svensk-intensivvard/.

[6] EMA. Treatments and vaccines for COVID-19 [cited 2021 January 24]. https://www.ema.europa.eu/en/human-regulatory/overview/public-health-threats/coronavirus-disease-covid-19/treatments-vaccines-covid-19.

[7] FDA. Coronavirus disease 2019 (COVID-19), Resources for Health Professionals: FDA; [cited 2021 January 24]. https://www.fda.gov/health-professionals/coronavirus-disease-2019-covid-19-resources-health-professionals.

[8] Jean S-S, Hsueh P-R. Old and re-purposed drugs for the treatment of COVID-19. Expert Rev Anti Infect Ther. 2020:1–5. doi: 10.1080/14787210.2020.1771181 32419524 PMC7441793

[9] Kamp TJ, Hamdan MH, January CT. Chloroquine or Hydroxychloroquine for COVID-19: Is Cardiotoxicity a Concern? J Am Heart Assoc. (2047–9980 (Electronic)). doi: 10.1161/JAHA.120.016887 32463308 PMC7429067

[10] Schrezenmeier E, Dorner T. Mechanisms of action of hydroxychloroquine and chloroquine: implications for rheumatology. Nat Rev Rheumatol. 2020;16(3):155–66. doi: 10.1038/s41584-020-0372-x 32034323

[11] Rainsford KD, Parke AL, Clifford-Rashotte M, Kean WF. Therapy and pharmacological properties of hydroxychloroquine and chloroquine in treatment of systemic lupus erythematosus, rheumatoid arthritis and related diseases. Inflammopharmacology. 2015;23(5):231–69. doi: 10.1007/s10787-015-0239-y 26246395

[12] Zhou D, Dai SM, Tong Q. COVID-19: a recommendation to examine the effect of hydroxychloroquine in preventing infection and progression. J Antimicrob Chemother. 2020;75(7):1667–70. doi: 10.1093/jac/dkaa114 32196083 PMC7184499

[13] Liu J, Cao R, Xu M, Wang X, Zhang H, Hu H, et al. Hydroxychloroquine, a less toxic derivative of chloroquine, is effective in inhibiting SARS-CoV-2 infection in vitro. Cell Discov. 2020;6:16. 10.1038/s41421-020-0156-0 PMC7078228 32194981

[14] Gao J, Tian Z, Yang X. Breakthrough: Chloroquine phosphate has shown apparent efficacy in treatment of COVID-19 associated pneumonia in clinical studies. Biosci Trends. 2020;14(1):72–3. doi: 10.5582/bst.2020.01047 32074550

[15] Gautret P, Lagier JC, Parola P, Hoang VT, Meddeb L, Sevestre J, et al. Clinical and microbiological effect of a combination of hydroxychloroquine and azithromycin in 80 COVID-19 patients with at least a six-day follow up: A pilot observational study. Travel Med Infect Dis. 2020:101663. doi: 10.1016/j.tmaid.2020.101663 32289548 PMC7151271

[16] Cipriani A, Zorzi A, Ceccato D, Capone F, Parolin M, Donato F, et al. Arrhythmic profile and 24-hour QT interval variability in COVID-19 patients treated with Hydroxychloroquine and azithromycin. Int J Cardiol. 2020;316:280–4. doi: 10.1016/j.ijcard.2020.05.036 32439366 PMC7235573

[17] Mercuro NJ, Yen CF, Shim DJ, Maher TR, McCoy CM, Zimetbaum PJ, et al. Risk of QT Interval Prolongation Associated With Use of Hydroxychloroquine With or Without Concomitant Azithromycin Among Hospitalized Patients Testing Positive for Coronavirus Disease 2019 (COVID-19). JAMA Cardiol. 2020;5(9):1036–41. doi: 10.1001/jamacardio.2020.1834 32936252 PMC7195692

[18] Mitja O, Corbacho-Monne M, Ubals M, Tebe C, Penafiel J, Tobias A, et al. Hydroxychloroquine for Early Treatment of Adults with Mild Covid-19: A Randomized-Controlled Trial. Clin Infect Dis. 2020;Epub ahead of print. doi: 10.1093/cid/ciaa1009 32674126 PMC7454406

[19] WHO. “Solidarity” clinical trial for COVID-19 treatments 2020 [https://www.who.int/emergencies/diseases/novel-coronavirus-2019/global-research-on-novel-coronavirus-2019-ncov/solidarity-clinical-trial-for-covid-19-treatments.

[20] Self WH, Semler MW, Leither LM, Casey JD, Angus DC, Brower RG, et al. Effect of Hydroxychloroquine on Clinical Status at 14 Days in Hospitalized Patients With COVID-19: A Randomized Clinical Trial. JAMA. 2020;324(21):2165–76. doi: 10.1001/jama.2020.22240 33165621 PMC7653542

[21] Molina JM, Delaugerre C, Le Goff J, Mela-Lima B, Ponscarme D, Goldwirt L, et al. No evidence of rapid antiviral clearance or clinical benefit with the combination of hydroxychloroquine and azithromycin in patients with severe COVID-19 infection. Med Mal Infect. 2020;50(4):384. doi: 10.1016/j.medmal.2020.03.006 32240719 PMC7195369

[22] Yazdany J, Kim AHJ. Use of Hydroxychloroquine and Chloroquine During the COVID-19 Pandemic: What Every Clinician Should Know. Ann Intern Med. 2020;172(11):754–5. doi: 10.7326/M20-1334 32232419 PMC7138336

[23] CllinicalTrials.gov: U.S. National Library of Medicine; [cited 2021 18 June]. https://clinicaltrials.gov/ct2/results?cond=Covid19 HYPERLINK "https://clinicaltrials.gov/ct2/results?cond=Covid19&term=chloroquine&cntry=&state=&city=&dist"& HYPERLINK "https://clinicaltrials.gov/ct2/results?cond=Covid19&term=chloroquine&cntry=&state=&city=&dist"term=chloroquine HYPERLINK "https://clinicaltrials.gov/ct2/results?cond=Covid19&term=chloroquine&cntry=&state=&city=&dist"& HYPERLINK "https://clinicaltrials.gov/ct2/results?cond=Covid19&term=chloroquine&cntry=&state=&city=&dist"cntry = HYPERLINK "https://clinicaltrials.gov/ct2/results?cond=Covid19&term=chloroquine&cntry=&state=&city=&dist"& HYPERLINK "https://clinicaltrials.gov/ct2/results?cond=Covid19&term=chloroquine&cntry=&state=&city=&dist"state = HYPERLINK "https://clinicaltrials.gov/ct2/results?cond=Covid19&term=chloroquine&cntry=&state=&city=&dist"& HYPERLINK "https://clinicaltrials.gov/ct2/results?cond=Covid19&term=chloroquine&cntry=&state=&city=&dist"city = HYPERLINK "https://clinicaltrials.gov/ct2/results?cond=Covid19&term=chloroquine&cntry=&state=&city=&dist"& HYPERLINK "https://clinicaltrials.gov/ct2/results?cond=Covid19&term=chloroquine&cntry=&state=&city=&dist"dist =.

[24] Vincent MJ, Bergeron E, Benjannet S, Erickson BR, Rollin PE, Ksiazek TG, et al. Chloroquine is a potent inhibitor of SARS coronavirus infection and spread. Virol J. 2005;2:69. doi: 10.1186/1743-422X-2-69 16115318 PMC1232869

[25] Paton NI, Lee L, Xu Y, Ooi EE, Cheung YB, Archuleta S, et al. Chloroquine for influenza prevention: a randomised, double-blind, placebo controlled trial. Lancet Infect Dis. 2011;11(9):677–83. doi: 10.1016/S1473-3099(11)70065-2 21550310

[26] Miller BR, Tsai TF, Mitchell CJ. Aedes aegypti and yellow fever virus: the effect of chloroquine on infection and transmission rates. Trans R Soc Trop Med Hyg. 1987;81(1):111–2. doi: 10.1016/0035-9203(87)90298-7 3445296

[27] Sperber K, Louie M, Kraus T, Proner J, Sapira E, Lin S, et al. Hydroxychloroquine treatment of patients with human immunodeficiency virus type 1. Clin Ther. 1995;17(4):622–36. doi: 10.1016/0149-2918(95)80039-5 8565026

[28] Chatre C, Roubille F, Vernhet H, Jorgensen C, Pers YM. Cardiac Complications Attributed to Chloroquine and Hydroxychloroquine: A Systematic Review of the Literature. Drug Saf. 2018;41(10):919–31. doi: 10.1007/s40264-018-0689-4 29858838

[29] Smeeth L, Thomas Sl Fau—Hall AJ, Hall Aj Fau—Hubbard R, Hubbard R Fau—Farrington P, Farrington P Fau—Vallance P, Vallance P. Risk of myocardial infarction and stroke after acute infection or vaccination. N Engl J Med. (1533–4406 (Electronic)). 10.1056/NEJMoa041747 15602021

[30] Corrales-Medina VF, Musher Dm Fau—Wells GA, Wells Ga Fau—Chirinos JA, Chirinos Ja Fau—Chen L, Chen L Fau—Fine MJ, Fine MJ. Cardiac complications in patients with community-acquired pneumonia: incidence, timing, risk factors, and association with short-term mortality. Circulation. (1524–4539 (Electronic)). doi: 10.1161/CIRCULATIONAHA.111.040766 22219349

[31] Zhou F, Yu T, Du R, Fan G, Liu Y, Liu Z, et al. Clinical course and risk factors for mortality of adult inpatients with COVID-19 in Wuhan, China: a retrospective cohort study. Lancet (London, England). 2020;395(10229):1054–62. doi: 10.1016/S0140-6736(20)30566-3 32171076 PMC7270627

[32] Efros O, Barda N, Meisel E, Leibowitz A, Fardman A, Rahav G, et al. Myocardial injury in hospitalized patients with COVID-19 infection-Risk factors and outcomes. PLoS One. 2021;16(2):e0247800. doi: 10.1371/journal.pone.0247800 33635914 PMC7909655

[33] Kraus D, von Jeinsen B, Tzikas S, Palapies L, Zeller T, Bickel C, et al. Cardiac Troponins for the Diagnosis of Acute Myocardial Infarction in Chronic Kidney Disease. J Am Heart Assoc. 2018;7(19):e008032. doi: 10.1161/JAHA.117.008032 30371308 PMC6404905

[34] Fan ZX, Yang J, Zhang J, He C, Wu H, Yang CJ, et al. Analysis of influencing factors related to elevated serum troponin I level for COVID-19 patients in Yichang, China. Cardiovasc Diagn Ther. 2020;10(4):678–86. doi: 10.21037/cdt-20-510 32968624 PMC7487375

[35] Arevalos V, Ortega-Paz L, Rodriguez-Arias JJ, Calvo M, Castrillo L, Salazar A, et al. Myocardial Injury in COVID-19 Patients: Association with Inflammation, Coagulopathy and In-Hospital Prognosis. J Clin Med. 2021;10(10). 10.3390/jcm10102096 PMC8152726 34068127

[36] Schiavone M, Gasperetti A, Mancone M, Kaplan AV, Gobbi C, Mascioli G, et al. Redefining the Prognostic Value of High-Sensitivity Troponin in COVID-19 Patients: The Importance of Concomitant Coronary Artery Disease. J Clin Med. 2020;9(10). doi: 10.3390/jcm9103263 33053826 PMC7601151

[37] Maraj I, Hummel JA-O, Taoutel R, Chamoun R, Workman V, Li C, et al. Incidence and Determinants of QT Interval Prolongation in COVID-19 Patients Treated with Hydroxychloroquine and Azithromycin. J Cardiovasc Electrophysiol. (1540–8167 (Electronic)). doi: 10.1111/jce.14594 32485061 PMC7300464

[38] Sinkeler FS, Berger FA, Muntinga HJ, Jansen M. The risk of QTc-interval prolongation in COVID-19 patients treated with chloroquine. Neth Heart J.28(7–8):418–23. doi: 10.1007/s12471-020-01462-6 32648153 PMC7346846

[39] Gasperetti A, Biffi M, Duru F, Schiavone M, Ziacchi M, Mitacchione G, et al. Arrhythmic safety of hydroxychloroquine in COVID-19 patients from different clinical settings. Europace. 2020;22(12):1855–63. doi: 10.1093/europace/euaa216 32971536 PMC7543547

[40] O’Connell TF, Bradley CJ, Abbas AE, Williamson BD, Rusia A, Tawney AM, et al. Hydroxychloroquine/Azithromycin Therapy and QT Prolongation in Hospitalized Patients With COVID-19. JACC Clin Electrophysiol. 2021;7(1):16–25. doi: 10.1016/j.jacep.2020.07.016 33478708 PMC7406234

